# Supplementary figures and images for: A thermodynamic bottleneck in the TCA cycle contributes to acetate overflow in Staphylococcus aureus
Source: mSphere. 2024 Dec 31;10(1):e00883-24. doi: 10.1128/msphere.00883-24 (PMC11774044; doi:10.1128/msphere.00883-24)

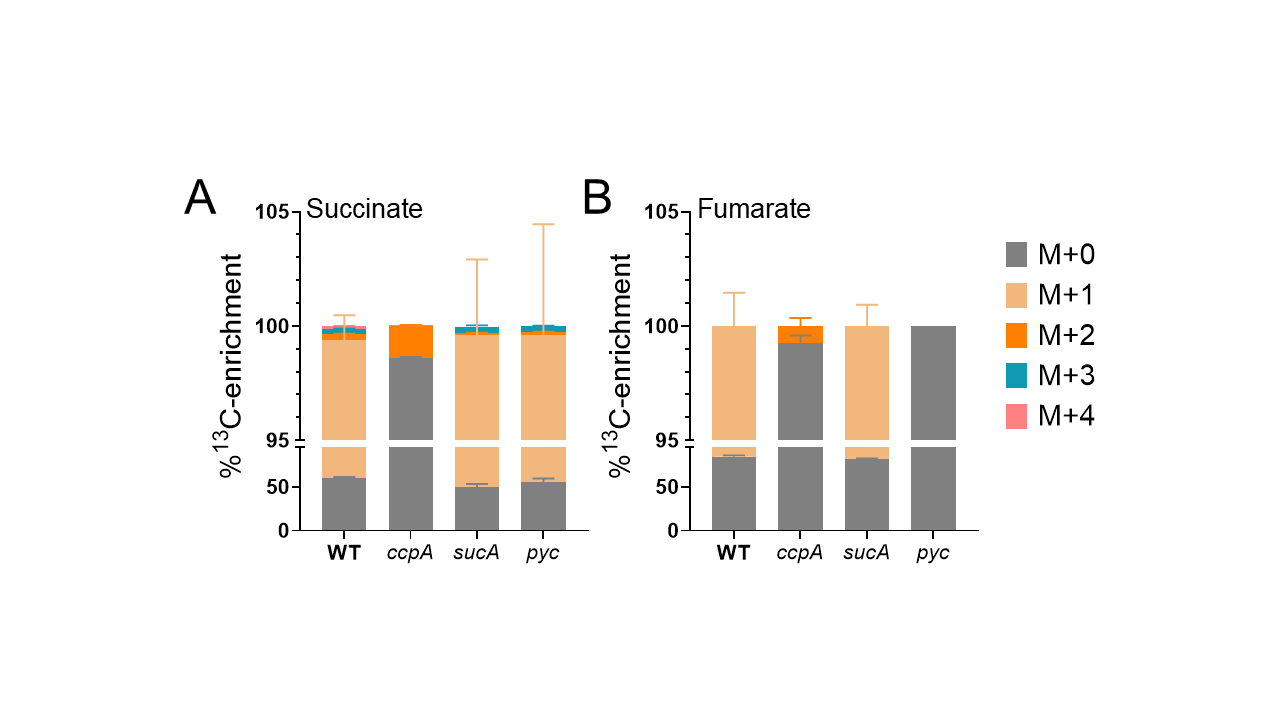

Supplement: Figure S1 — Mass isotopologueg distribution of metabolites. [file msphere.00883-24-s0006.tif]
