# Supplementary material for: A thermodynamic bottleneck in the TCA cycle contributes to acetate overflow in Staphylococcus aureus
Source: mSphere. 2024 Dec 31;10(1):e00883-24. doi: 10.1128/msphere.00883-24 (PMC11774044; doi:10.1128/msphere.00883-24)
Supplement: Supplemental text — Supplemental materials and methods and Fig. S1 legend. [file msphere.00883-24-s0007.docx]

**Supplementary Material**

**Material and Methods**

**Growth analysis**

For growth analysis, *S. aureus* cultures were grown aerobically at 37°C in 36 ml TSB supplemented with 14 mM glucose (in 250-ml Erlenmeyer flasks) for 9 h. We selected 14 mM glucose based on prior studies to ensure conditions sufficient for inducing overflow metabolism and to promote rapid growth. Culture aliquots of 1 ml were collected every 1.5 hours, and optical density at wavelength of 600 nm was measured spectrophotometrically.

**Extracellular metabolite analysis**

For extracellular metabolite analysis, 1 ml aliquots of bacterial cultures were collected at the indicated time points and centrifuged at 16,000 × g for 3 minutes. The supernatants were collected and stored at −20°C for further analysis. Glucose, lactate, and acetate concentrations in culture supernatants were determined using commercial kits (R-Biopharm), according to the manufacturer's instructions.

**Sample collection for mass-spectrometry analysis**

Overnight cultures of WT (JE2 strain), *ccpA*, *sucA*, and *pyc* mutants were inoculated to an OD_600_ of 0.06 units into 250 ml flasks containing 25ml of TSB 14 mM U-^13^C_6_-D-glucose. The flasks were incubated in a shaker incubator at 37°C and 250 rpm. Upon reaching the exponential phase (after 2 hours and 15 minutes), a culture volume corresponding to a total of 10 OD_600_ units of cells was rapidly filtered through a 0.22 µm pore-size filter. Bacteria retained on the filter were washed twice with ice-cold saline (0.6% NaCl) and then resuspended in an ice-cold quenching solution consisting of 60% ethanol and 2 µM ribitol, used as an internal standard. The cytosolic metabolites were obtained by bead beating the cells, followed by centrifugation. The supernatant was collected, lyophilized and stored at -80 °C until required. Lyophilized samples were reconstituted in 100 µl of 50% MeOH prior to LC-MS/MS analysis.

**LC-MS/MS analysis**

Triple-quadrupole-ion trap hybrid mass spectrometer viz., QTRAP6500+ (Sciex, USA) connected with ultra-performance liquid chromatography *I*-class (UPLC) system procured from Waters, USA was used for the metabolite analysis. The chromatographic separation was performed by liquid chromatography using XBridge C18 (100 × 2.1mm ID; 1.7µm particle size, Waters, USA) analytical column and a binary solvent system with a flow rate of 0.1 ml/min. A guard XBridge C18 column (20 × 2.1mm ID; 1.7µm particle size, Waters, USA) was connected in front of analytical column. Mobile phase A was composed of 0.1% formic acid LC-MS grade water; mobile phase B was 100% LC-MS grade acetonitrile. The column was maintained at 40°C and autosampler temperature was maintained at 5°C. The gradient was started with the A/B solvent ratio at 95/5 which was maintained for 1.0 minute followed by a gradual increase of B to 50% for 5.0 minute. B was further increased to 90% for 3.0 minutes and maintained at 90% over 2.0 minutes then decreased to 5% over next 0.5 minute. The column was equilibrated at this A/B ratio for next 5.5 minutes before the next run. 500µl Strong wash solvent for needle containing 100% acetonitrile whereas 500µl of weak wash solvent comprised of 10% aqueous methanol were used after each injection. Injection volume was 5µl. QTRAP6500+ operated in polarity switching mode was used for targeted quantitation of metabolites and their respective isotopologues through Multiple Reaction Monitoring (MRM) process. Electrospray ionization (ESI) parameters were optimized as follows: electrospray ion voltage of -4200V and 5500V in negative and positive mode respectively, source temperature of 400°C, curtain gas of 35, and gas 1 and 2 of 40 and 40 psi, respectively. Compound specific parameters such as declustering potential (DP), entrance potential (EP), collision cell exit potential (CXP) and collision energy were optimized for un-labeled standards using manual tuning. These parameters were adopted for isotopologue of each metabolite of interest. To correct for natural abundance, we utilized FluxFix (1), an open-source online software, and independently verified these calculations using the ChemCalc software (2).

| **Metabolite** | **Isotopologue** | **MRM (Q1/Q3) m/z** | **Collision Energy (V)** |
| --- | --- | --- | --- |
| Fumarate | M+0 | 115.0 / 71.0 | -13 |
|  | M+1 | 116.0 / 71.0 | -13 |
|  | M+2 | 117.0 / 72.0 | -13 |
|  | M+3 | 120.0/102.0 | -13 |
|  | M+4 | 121.0/103.0 | 13 |
| Succinate | M+0 | 117.0 / 73.0 | -14 |
|  | M+1 | 118.0 / 74.0 | -14 |
|  | M+2 | 119.0 / 74.0 | -14 |
|  | M+3 | 120.0 / 76.0 | -14 |
|  | M+4 | 121.0 / 76.0 | -14 |

**Refinement and integration of omics data into genome-scale metabolic model.**

To create a more accurate and reliable representation of *S. aureus* metabolism, extensive curation was performed on the initial genome-scale metabolic (GSM) model (3). The goal of this refinement was to achieve a high consistency score by improving the number of mass and charge-balanced reactions. The MEMOTE score (4) was used to quantify the quality and consistency of the model. Inconsistencies in the initial model were addressed by correcting mass imbalanced reactions and standardizing the biomass reaction. Furthermore, inaccuracies in the charged formulas of certain metabolites were resolved to ensure that all reactions were both stoichiometrically and thermodynamically consistent.

| **Consistency** | **Score** |
| --- | --- |
| Stoichiometric Consistency | 100% |
| Mass Balance | 99.2% |
| Charge Balance | 87.5 |
| Metabolite Connectivity | 100% |
| Unbounded | 94.3% |

For the integration of omics data, three algorithms—iMAT (5), RIPTiDe (6), EXTREAM (7) and E-Flux (8)—were applied to contextualize the model based on experimental data, minimizing biases. Nutrient uptakes, including glucose and essential amino acids (alanine, valine, serine, leucine, lysine, proline, glycine, isoleucine, threonine, aspartic acid, glutamate, glutamine, phenylalanine, asparagine, arginine, and cysteine), were adjusted to reflect the experimental nutrient conditions. The contextualized models generated by these algorithms were then compared with experimental data to assess their accuracy in replicating the organism's behavior.

To evaluate the models, we used metabolomics data to calculate the error percentage between the computational model predictions and experimental results for the ratio of acetate secretion to glucose uptake. The error percentage was determined using the following formula:

$$Error \%=\left( \frac{Ratio from contextual model-Ratio from metabolomics}{Ratio from metabolomics} \right)\times100$$

$Here,$ $Ratio=\frac{acetate secretion}{glucose uptake}$

**Integration of ‘InteGraM’ in OptMDFpathway**

In our exploration of the metabolic network of *S. aureus*, we utilized the OptMDFpathway(9) method, integrated with contextualized GEMs and thermodynamic data. Thermodynamic data for reactions were sourced from the 'equilibrator' database (10). Of the 1560 reactions represented in the model, 227 were exchange reactions, and thermodynamic driving force data was available for more than 65% of the 1333 internal reactions (see **Supplementary Data 4**). The constraints for metabolite concentrations were defined following the max-min driving force (MDF) analysis (11) equations (1–8).

Furthermore, the InteGraM algorithm was incorporated as an enhancement within the OptMDFpathway framework. This method aimed to maximize the minimum driving force (B) across the metabolic network using a set of constraints formulated as a mixed-integer linear programming (MILP) problem. The objective function and constraints are defined as follows: $Maximize B$

$$subject to$$

$$\sum_{j\in J} S_{ij}.v_{j}=0 \forall i\in I \left( 1 \right)$$

$${LB}_{j}\leq v_{j}\leq{UB}_{j} \forall j\in J \left( 2 \right)$$

$$Dv\leq d \forall j\in J \left( 3 \right)$$

$$Dv\leq d \left( 4 \right)$$

$f_{j}= {-\Delta}_{v}G_{j}^{'}= =\left( \Delta_{v}G_{j}^{'0}+RT. S_{ij}^{T} .x \right) (5)$

$$\ln\left( C_{min} \right)\leq x \leq\ln\left( C_{max} \right) \left( 6 \right)$$

$$v_{j}\leq{z_{j} . UB}_{j} (7)$$

$$f_{j}+\left( 1+ z_{j} \right). M_{j} \geq B (8)$$

$$\sum_{m\in J} {SASA}_{m}.v_{m}=c \forall m\in J \left( 9 \right)$$

$$SASA= {(molecular weight of the enzyme)}^{\frac{3}{4}} (10)$$

Here, *I* and *J* are the sets of metabolites and reactions in the model, respectively. *S_ij_* is the stoichiometric coefficient of metabolite *i* in reaction *j* and *v_j_* is the flux value of reaction *j*. Parameters *LB_j_* and *UB_j_* denote the minimum and maximum allowable fluxes for reaction *j*, respectively. $\Delta_{v}G_{j}^{'0}$is a vector containing the standard change in Gibbs energy of the involved reactions, $C_{min}$and $C_{max}$are the vectors of metabolite concentration limits, $x$is the vector of logarithmized metabolite concentrations and $RT$ is the product of the universal gas constant. Also, *m* represents the membrane-bound reactions, *c* represents the maximum sum of membrane-bound reactions’ fluxes. Equations (1) through (8) establish the foundational framework for assessing metabolic pathway efficiency, integrating thermodynamic data to predict reaction feasibility. Equation (9) specifically explores the membrane crowding effect and so a new constraint is added in the formulation. Here, the term SASA stands for surface accessible surface area which is calculated based on molecular weight of enzymes (equation 10). This value is then normalized by the sum of all membrane-bound enzymes' SASA and multiplied by the reaction flux to obtain the final result. Following this, the value of *B* is determined based on the constraints. Once *B* is fixed, the maximum and minimum values of the multiplication factor are calculated, which are subsequently used to determine the acetate levels.

**Supplementary Fig. 1** Mass isotopologue distribution (MID) of metabolites.

**References**

1. Trefely S, Ashwell P, Snyder NW. 2016. FluxFix: automatic isotopologue normalization for metabolic tracer analysis. BMC Bioinformatics 17:485.

2. Patiny L, Borel A. 2013. ChemCalc: A Building Block for Tomorrow’s Chemical Infrastructure. J Chem Inf Model 53:1223–1228.

3. Mazharul Islam M, Thomas VC, Van Beek M, Ahn JS, Alqarzaee AA, Zhou C, Fey PD, Bayles KW, Saha R. 2020. An integrated computational and experimental study to investigate Staphylococcus aureus metabolism. NPJ Syst Biol Appl 6:1–13.

4. Lieven C, Beber ME, Olivier BG, Bergmann FT, Ataman M, Babaei P, Bartell JA, Blank LM, Chauhan S, Correia K, Diener C, Dräger A, Ebert BE, Edirisinghe JN, Faria JP, Feist AM, Fengos G, Fleming RMT, García-Jiménez B, Hatzimanikatis V, van Helvoirt W, Henry CS, Hermjakob H, Herrgård MJ, Kaafarani A, Kim HU, King Z, Klamt S, Klipp E, Koehorst JJ, König M, Lakshmanan M, Lee DY, Lee SY, Lee S, Lewis NE, Liu F, Ma H, Machado D, Mahadevan R, Maia P, Mardinoglu A, Medlock GL, Monk JM, Nielsen J, Nielsen LK, Nogales J, Nookaew I, Palsson BO, Papin JA, Patil KR, Poolman M, Price ND, Resendis-Antonio O, Richelle A, Rocha I, Sánchez BJ, Schaap PJ, Malik Sheriff RS, Shoaie S, Sonnenschein N, Teusink B, Vilaça P, Vik JO, Wodke JAH, Xavier JC, Yuan Q, Zakhartsev M, Zhang C. 2020. MEMOTE for standardized genome-scale metabolic model testing. Nat Biotechnol 38:272–276.

5. Zur H, Ruppin E, Shlomi T. 2010. iMAT: An integrative metabolic analysis tool. Bioinformatics 26:3140–3142.

6. Jenior ML, Moutinho TJ, Dougherty B V., Papin JA. 2020. Transcriptome-guided parsimonious flux analysis improves predictions with metabolic networks in complex environments. PLoS Comput Biol 16.

7. Chowdhury NB, Simons-Senftle M, Decouard B, Quillere I, Rigault M, Sajeevan KA, Acharya B, Chowdhury R, Hirel B, Dellagi A, Maranas C, Saha R. 2023. A multi-organ maize metabolic model connects temperature stress with energy production and reducing power generation. iScience 26:108400.

8. Colijn C, Brandes A, Zucker J, Lun DS, Weiner B, Farhat MR, Cheng TY, Moody DB, Murray M, Galagan JE. 2009. Interpreting expression data with metabolic flux models: Predicting Mycobacterium tuberculosis mycolic acid production. PLoS Comput Biol 5.

9. Hädicke O, von Kamp A, Aydogan T, Klamt S. 2018. OptMDFpathway: Identification of metabolic pathways with maximal thermodynamic driving force and its application for analyzing the endogenous CO2 fixation potential of Escherichia coli. PLoS Comput Biol 14:e1006492.

10. Beber ME, Gollub MG, Mozaffari D, Shebek KM, Flamholz AI, Milo R, Noor E. 2022. eQuilibrator 3.0: a database solution for thermodynamic constant estimation. Nucleic Acids Res 50:D603–D609.

11. Noor E, Bar-Even A, Flamholz A, Reznik E, Liebermeister W, Milo R. 2014. Pathway Thermodynamics Highlights Kinetic Obstacles in Central Metabolism. PLoS Comput Biol 10.
